# Supplementary material for: Pathogenic and genomic characterization of rabbit-sourced Pasteurella multocida serogroup F isolates recovered from dead rabbits with respiratory disease
Source: Microbiol Spectr. 2024 Feb 22;12(4):e03654-23. doi: 10.1128/spectrum.03654-23 (PMC10986509; doi:10.1128/spectrum.03654-23)
Supplement: Figure S1 — Pathologic and histopathological lesions in the white feather broilers. [file spectrum.03654-23-s0001.pdf]

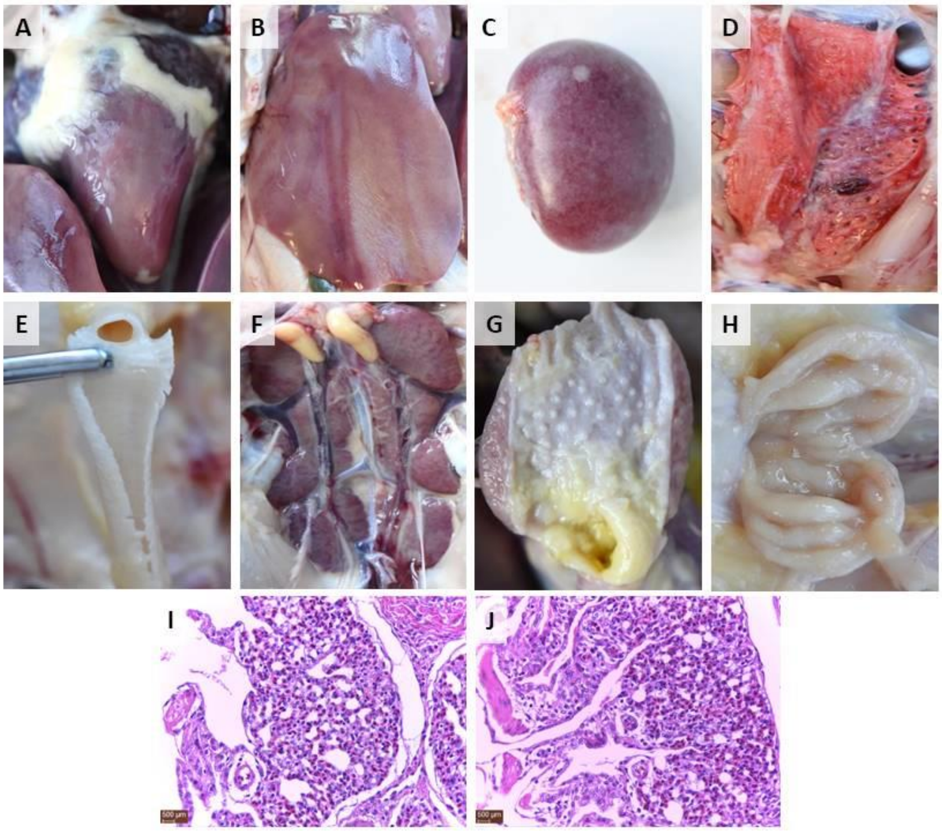

Fig S1 The pathologic and histopathological lesions in the white feather broilers intratracheally inoculated with the 19 isolates. There were no pathologic and histopathological lesions observed in the white feather broilers intratracheally inoculated with the 19 isolates. A: heart; B: liver; C: spleen; D: lung; E: trachea; F: kidney; G: ventriculus glandularis; H: bursa of Fabricius; I: lung section from the white feather broilers intratracheally inoculated with the 19 isolates; J: lung section from the white feather broilers intratracheally inoculated with the sterile normal saline;
