# Supplementary material for: Pathogenic and genomic characterization of rabbit-sourced Pasteurella multocida serogroup F isolates recovered from dead rabbits with respiratory disease
Source: Microbiol Spectr. 2024 Feb 22;12(4):e03654-23. doi: 10.1128/spectrum.03654-23 (PMC10986509; doi:10.1128/spectrum.03654-23)
Supplement: Figure S2 — Circular maps of the plasmid of PF1 and PF13. [file spectrum.03654-23-s0002.pdf]

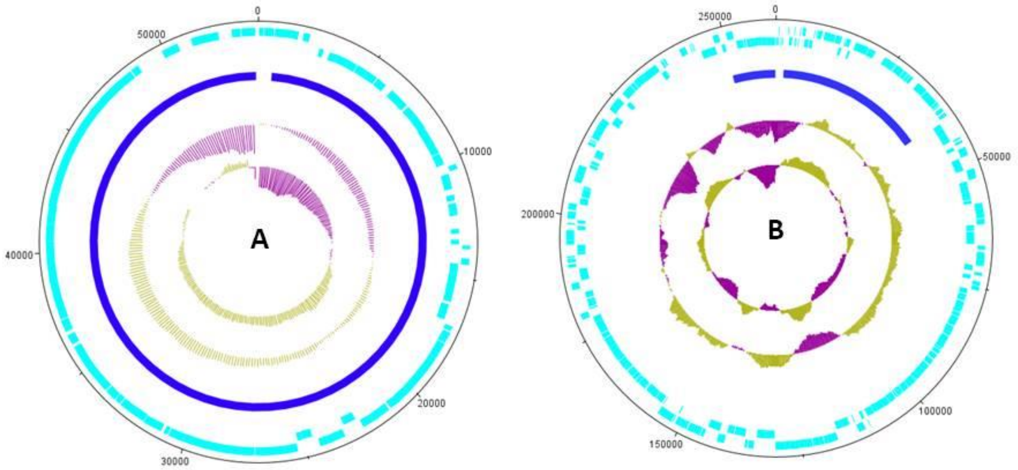

Fig S2 Circular maps of the plasmid of PF1 and PF13. A: plasmid of PF1; B: plasmid of PF13. From the outside to the inside, circle 1 (black): DNA base position; circle 2 (cyan-blue): protein-coding regions in forward strand; circle 3 (cyan-blue): protein-coding regions in reverse strand; circle 4 (blue): prophage sequence; the two innermost circles represent the G＋C content and GC skew, respectively.
