# Supplementary material for: Pathogenic and genomic characterization of rabbit-sourced Pasteurella multocida serogroup F isolates recovered from dead rabbits with respiratory disease
Source: Microbiol Spectr. 2024 Feb 22;12(4):e03654-23. doi: 10.1128/spectrum.03654-23 (PMC10986509; doi:10.1128/spectrum.03654-23)
Supplement: Figure S3 — Colinear analyses of the genomes between the 19 isolates and that of s4. [file spectrum.03654-23-s0003.pdf]

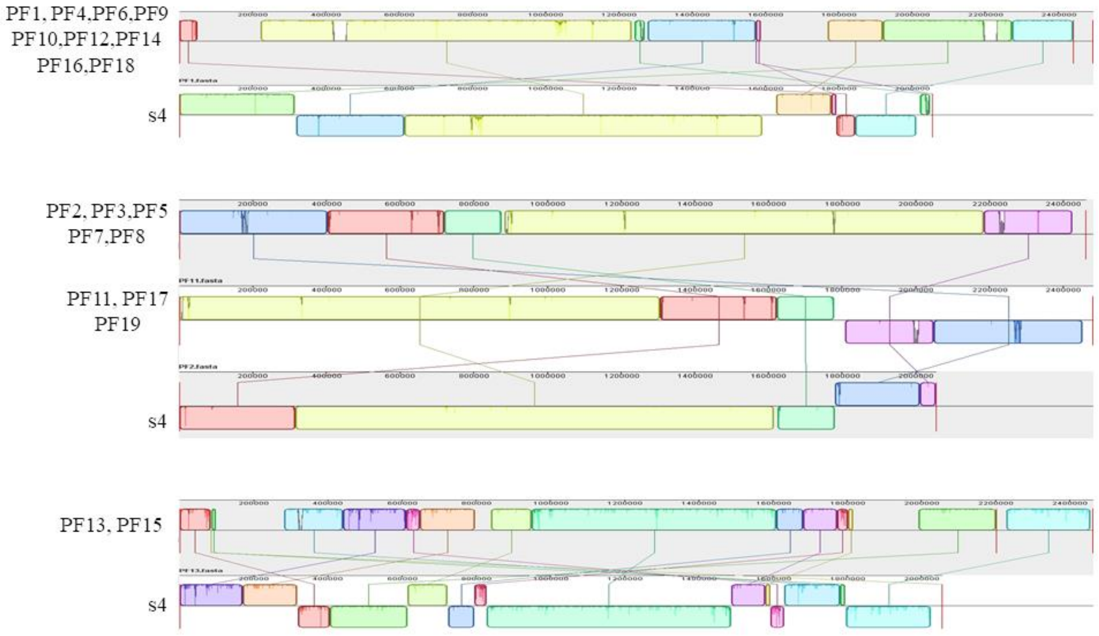

Fig S3 Colinear analyses of the genomes between the 19 isolates and that of s4. The genomes of the 19 isolates showed highly collinear with that of s4, and three collinear patterns were determined between the genomes of the 19 isolates and that of s4 corresponding to the phylogenetic analysis result. Rectangle of the same colour indicates the similar local collinear between the 19 isolates and s4.
