# Supplementary figures and images for: Pathogenic and genomic characterization of rabbit-sourced Pasteurella multocida serogroup F isolates recovered from dead rabbits with respiratory disease
Source: Microbiol Spectr. 2024 Feb 22;12(4):e03654-23. doi: 10.1128/spectrum.03654-23 (PMC10986509; doi:10.1128/spectrum.03654-23)

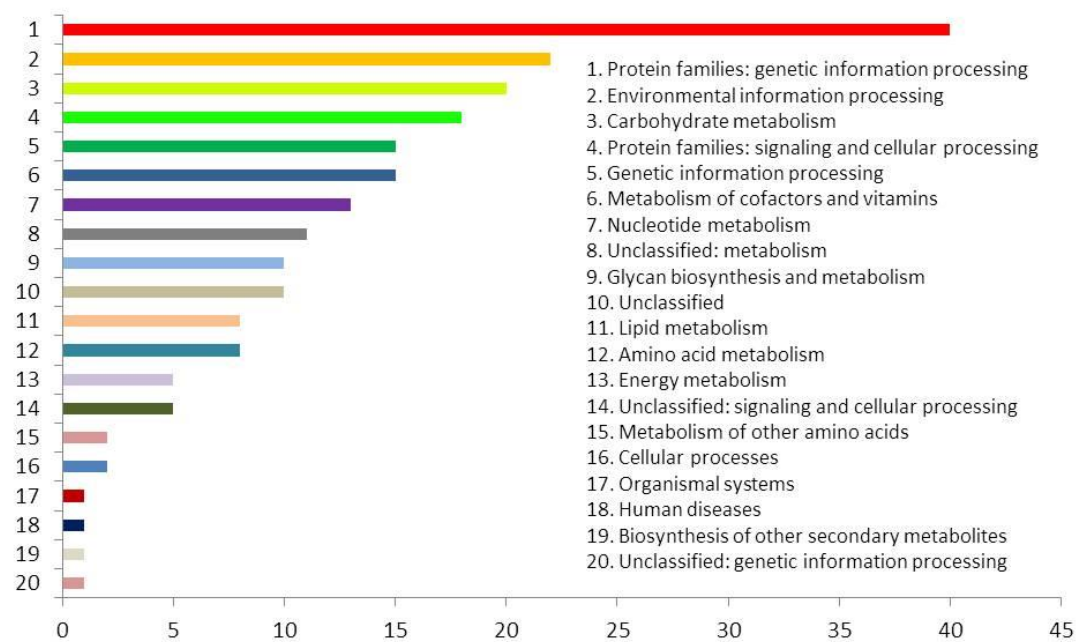

**Fig S4** The functional genes identified in the genome of the 19 isolates but absent in that of s4.

Supplement: Figure S4 — Functional genes identified in the genomes of the 19 isolates but absent in that of s4. [file spectrum.03654-23-s0004.pdf]
