# Supplementary material for: Pathogenic and genomic characterization of rabbit-sourced Pasteurella multocida serogroup F isolates recovered from dead rabbits with respiratory disease
Source: Microbiol Spectr. 2024 Feb 22;12(4):e03654-23. doi: 10.1128/spectrum.03654-23 (PMC10986509; doi:10.1128/spectrum.03654-23)
Supplement: Table S1 — Details of the isolates. [file spectrum.03654-23-s0005.docx]

**Table S1** Details of the 19 isolates used in this study and results of capsular, LPS and multi-locus sequence typing

| Region | Strain | Lesion | Capsule genotype | LPS genotype | Multi-host MLST |
| --- | --- | --- | --- | --- | --- |
| Fuzhou | PF1 | Fibrinopurulent pleuropneumonia | F | L3 | ST191 |
|  | PF2 | Fibrino pleuropneumonia | F | L3 | ST12 |
|  | PF3 | Pulmonary consolidation with hemorrhagic pneumonia | F | L3 | ST12 |
|  | PF4 | Fibrino pleuropneumonia | F | L3 | ST191 |
| Longyan | PF5 | Pulmonary consolidation with hemorrhagic pneumonia | F | L3 | ST12 |
|  | PF6 | Fibrinopurulent pleuropneumonia | F | L3 | ST191 |
|  | PF7 | Fibrino pleuropneumonia | F | L3 | ST12 |
|  | PF8 | Fibrino pleuropneumonia | F | L3 | ST12 |
|  | PF9 | Fibrinopurulent pleuropneumonia | F | L3 | ST191 |
|  | PF10 | Fibrinopurulent pleuropneumonia | F | L3 | ST191 |
|  | PF11 | Fibrino pleuropneumonia | F | L3 | ST193 |
|  | PF12 | Fibrinopurulent pleuropneumonia | F | L3 | ST191 |
|  | PF13 | Pulmonary consolidation with hemorrhagic pneumonia | F | L3 | ST192 |
|  | PF14 | Pulmonary consolidation with hemorrhagic pneumonia | F | L3 | ST191 |
| Nanping | PF15 | Fibrino pleuropneumonia | F | L3 | ST192 |
|  | PF16 | fibrinopurulent pleuropneumonia | F | L3 | ST191 |
|  | PF17 | Fibrino pleuropneumonia | F | L3 | ST193 |
| Sanming | PF18 | Fibrinopurulent pleuropneumonia | F | L3 | ST191 |
|  | PF19 | Pulmonary consolidation with hemorrhagic pneumonia | F | L3 | ST193 |
