# Supplementary material for: Pathogenic and genomic characterization of rabbit-sourced Pasteurella multocida serogroup F isolates recovered from dead rabbits with respiratory disease
Source: Microbiol Spectr. 2024 Feb 22;12(4):e03654-23. doi: 10.1128/spectrum.03654-23 (PMC10986509; doi:10.1128/spectrum.03654-23)
Supplement: Table S2 — Numbers of rabbits euthanized and survived. [file spectrum.03654-23-s0006.docx]

**Table S2** Numbers of rabbits euthanized and survived during the 15-day experiment period

| Isolates | Subcutaneous inoculation | | | Intranasal inoculation | | |
| --- | --- | --- | --- | --- | --- | --- |
|  | Euthanasia | Survived | Mortality (%) | Euthanasia | Survived | Mortality (%) |
| PF1 | 6 | 2 | 75** | 4 | 4 | 50* |
| PF2 | 5 | 3 | 62.5* | 5 | 3 | 62.5* |
| PF3 | 4 | 4 | 50* | 4 | 4 | 50* |
| PF4 | 7 | 1 | 87.5** | 5 | 3 | 62.5* |
| PF5 | 5 | 3 | 62.5* | 3 | 5 | 37.5 |
| PF6 | 6 | 2 | 75** | 6 | 2 | 75** |
| PF7 | 5 | 3 | 62.5* | 5 | 3 | 62.5* |
| PF8 | 6 | 2 | 75** | 5 | 3 | 62.5* |
| PF9 | 6 | 2 | 75** | 6 | 2 | 75** |
| PF10 | 7 | 1 | 87.5** | 5 | 3 | 62.5* |
| PF11 | 4 | 4 | 50* | 5 | 3 | 62.5* |
| PF12 | 7 | 1 | 87.5** | 6 | 2 | 75** |
| PF13 | 4 | 4 | 50* | 3 | 5 | 37.5 |
| PF14 | 6 | 2 | 75** | 6 | 2 | 75** |
| PF15 | 4 | 4 | 50* | 3 | 5 | 37.5 |
| PF16 | 7 | 1 | 87.5** | 6 | 2 | 75** |
| PF17 | 5 | 3 | 62.5* | 3 | 5 | 37.5 |
| PF18 | 7 | 1 | 87.5** | 6 | 2 | 75** |
| PF19 | 4 | 4 | 50* | 3 | 5 | 37.5 |
| Control | 0 | 8 | 0 | 0 | 8 | 0 |

Statistically significant differences versus control rabbits are shown (*P＜0.05 and **P＜0.01)
