# Supplementary material for: Pathogenic and genomic characterization of rabbit-sourced Pasteurella multocida serogroup F isolates recovered from dead rabbits with respiratory disease
Source: Microbiol Spectr. 2024 Feb 22;12(4):e03654-23. doi: 10.1128/spectrum.03654-23 (PMC10986509; doi:10.1128/spectrum.03654-23)
Supplement: Table S3 — Genome overview of the isolates. [file spectrum.03654-23-s0007.docx]

**Table S3** Genome overview of the 19 isolates

| Strain | No. of reads | Coverage (×) | *N_50_* value | Total sequence length (bp) | G+C content (%) | No. of genes | No. of proteins | GenBank accession no. |
| --- | --- | --- | --- | --- | --- | --- | --- | --- |
| PF1 | 403,740 | 838.0 | 9,701 | 2,495,990 | 40.32 | 2,460 | 2,354 | CP112898, CP112899 |
| PF2 | 167,086 | 717.0 | 11,836 | 2,482,587 | 40.35 | 2,446 | 2,322 | CP111081 |
| PF3 | 60,176 | 382.0 | 20,340 | 2,461,534 | 40.34 | 2,409 | 2,302 | CP111082 |
| PF4 | 177,192 | 887.0 | 19,806 | 2,495,991 | 40.32 | 2,460 | 2,355 | CP111083 |
| PF5 | 65,963 | 315.0 | 14,992 | 2,461,710 | 40.34 | 2,412 | 2,303 | CP111142 |
| PF6 | 128,439 | 796.0 | 21,834 | 2,495,991 | 40.32 | 2,460 | 2,355 | CP111143 |
| PF7 | 55,097 | 301.0 | 17,357 | 2,461,707 | 40.34 | 2,410 | 2,301 | CP111144 |
| PF8 | 62,443 | 349.0 | 18,140 | 2,461,504 | 40.34 | 2,410 | 2,303 | CP113236 |
| PF9 | 144,204 | 549.0 | 12,736 | 2,496,043 | 40.32 | 2,464 | 2,349 | CP111145 |
| PF10 | 172,158 | 721.0 | 13,295 | 2,496,048 | 40.32 | 2,465 | 2,345 | CP111146 |
| PF11 | 77,771 | 207.0 | 11,549 | 2,461,682 | 40.34 | 2,426 | 2,299 | CP111147 |
| PF12 | 173,395 | 695.0 | 13,131 | 2,496,027 | 40.32 | 2,470 | 2,350 | CP112891 |
| PF13 | 191,912 | 737.0 | 12,000 | 2,463,826 | 40.30 | 2,419 | 2,303 | CP113522, CP113523 |
| PF14 | 150,319 | 580.0 | 12,655 | 2,496,037 | 40.32 | 2,466 | 2,345 | CP112892 |
| PF15 | 141,083 | 554.0 | 12,312 | 2,463,975 | 40.30 | 2,416 | 2,300 | CP112893 |
| PF16 | 108,324 | 409.0 | 12,387 | 2,496,019 | 40.32 | 2,465 | 2,348 | CP112894 |
| PF17 | 66,460 | 269.0 | 12,938 | 2,461,646 | 40.34 | 2,417 | 2,288 | CP112895 |
| PF18 | 148,624 | 572.0 | 12,077 | 2,496,019 | 40.32 | 2,473 | 2,356 | CP112896 |
| PF19 | 102,579 | 400.0 | 12,410 | 2,461,989 | 40.34 | 2,416 | 2,305 | CP112897 |
