# Supplementary material for: Pathogenic and genomic characterization of rabbit-sourced Pasteurella multocida serogroup F isolates recovered from dead rabbits with respiratory disease
Source: Microbiol Spectr. 2024 Feb 22;12(4):e03654-23. doi: 10.1128/spectrum.03654-23 (PMC10986509; doi:10.1128/spectrum.03654-23)
Supplement: Table S4 — Prophage sequences and gnomic islands of the isolates. [file spectrum.03654-23-s0008.docx]

**Table S4** Prophage sequences and gnomic islands in the genomes of the 19 isolates

| Isolate | Prophage | | | | Genomic island | | |
| --- | --- | --- | --- | --- | --- | --- | --- |
|  | Location | Size (Kb) | Coppleteness | Region position | Location | Size (Kb) | Region position |
| PF1 | Chr | 48.9 | intact | 45,656-94,588 | Chr | 47.4 | 46,852-94,232 |
|  | Chr | 36.6 | intact | 419,508-456,110 | Chr | 37.0 | 419,371-456,414 |
|  |  |  |  |  | Chr | 5.8 | 589,224-595,051 |
|  | Chr | 34.4 | intact | 1,020,662-1,055,098 | Chr | 35.9 | 1,020,162-1,056,041 |
|  | Chr | 43.2 | intact | 1,237,020-1,280,198 | Chr | 44.9 | 1,235,327-1,280,198 |
|  |  |  |  |  | Chr | 14.1 | 1,571,449-1,585,563 |
|  |  |  |  |  | Chr | 5.4 | 1,756,956-1,762,381 |
|  | Chr | 50.9 | intact | 2,195,624-2,246,526 | Chr | 12.0 | 2,196,797-2,208,801 |
|  |  |  |  |  | Chr | 7.1 | 2,218,428-2,225,537 |
|  | P | 53.1 | intact | 684-53,820 |  |  |  |
| PF2 | Chr | 33.9 | intact | 65-33,970 | Chr | 5.4 | 684-6,120 |
|  |  |  |  |  | Chr | 7.1 | 15,762-22,871 |
|  |  |  |  |  | Chr | 5.8 | 359,351-365,178 |
|  | Chr | 36.2 | intact | 790,815-827,039 | Chr | 36.5 | 790,315-826,782 |
|  | Chr | 46.5 | intact | 1,771,899-1,818,384 | Chr | 12.0 | 1,775,333-1,787,339 |
|  |  |  |  |  | Chr | 10.0 | 1,820,256-1,830,287 |
|  | Chr | 42.1 | intact | 2,007,622-2,049,765 | Chr | 53.3 | 1,995,455-2,048,776 |
|  | Chr | 32.7 | intact | 2,290,987-2,323,689 | Chr | 64.4 | 2,263,766-2,328,168 |
|  | Chr | 28.8 | intact | 2,453,401-2,482,157 | Chr | 21.4 | 2,446,465-2,467,851 |
| PF3 | Chr | 32.7 | intact | 73,962-106,656 | Chr | 61.6 | 46,735-108,305 |
|  | Chr | 38.7 | intact | 236,368-275,050 | Chr | 45.0 | 229,432-274,400 |
|  |  |  |  |  | Chr | 5.8 | 604,053-609,880 |
|  | Chr | 36.2 | intact | 1,035,517-1,071,741 | Chr | 36.5 | 1,035,017-1,071,484 |
|  | Chr | 67.5 | intact | 2,005,967-2,073,491 | Chr | 4.5 | 2,012,744-2,017,216 |
|  |  |  |  |  | Chr | 39.1 | 2,017,595-2,056,731 |
|  |  |  |  |  | Chr | 13.5 | 2,061,517-2,074,994 |
|  | Chr | 42.1 | intact | 2,252,392-2,294,535 | Chr | 54.3 | 2,240,222-2,294,535 |
| PF4 | Chr | 48.9 | intact | 45,656-94,588 | Chr | 47.4 | 46,852-94,232 |
|  | Chr | 36.6 | intact | 419,508-456,110 | Chr | 37.0 | 419,371-456,414 |
|  |  |  |  |  | Chr | 5.8 | 589,224-595,051 |
|  | Chr | 34.4 | intact | 1,020,662-1,055,098 | Chr | 11.2 | 1,020,162-1,031,368 |
|  | Chr | 49.6 | intact | 1,230,648-1,280,198 | Chr | 44.9 | 1,235,327-1,280,198 |
|  |  |  |  |  | Chr | 14.1 | 1,571,449-1,585,563 |
|  | Chr | 67.6 | intact | 1,751,919-1,819,502 | Chr | 66.0 | 1,749,922-1,815,936 |
|  |  |  |  |  | Chr | 4.1 | 1,815,706-1,819,775 |
|  | Chr | 50.9 | intact | 2,249,571-2,300,473 | Chr | 37.0 | 2,249,287-2,286,319 |
| PF5 | Chr | 32.7 | intact | 74,228-106,931 | Chr | 61.6 | 47,001-108,580 |
|  | Chr | 38.6 | intact | 236,604-275,286 | Chr | 21.4 | 229,668-274,636 |
|  |  |  |  |  | Chr | 5.8 | 604,289-610,116 |
|  | Chr | 36.2 | intact | 1,035,753-1,071,977 | Chr | 36.5 | 1,035,253-1,071,720 |
|  | Chr | 67.5 | intact | 2,006,203-2,073,727 | Chr | 5.1 | 2,012,340-2,017,452 |
|  |  |  |  |  | Chr | 39.1 | 2,017,831-2,056,967 |
|  |  |  |  |  | Chr | 10.0 | 2,065,199-2,075,230 |
|  | Chr | 42.1 | intact | 2,252,568-2,294,711 | Chr | 54.3 | 2,240,398-2,294,711 |
| PF6 | Chr | 48.9 | intact | 45,656-94,588 | Chr | 47.4 | 46,852-94,232 |
|  | Chr | 36.6 | intact | 419,508-456,110 | Chr | 37.0 | 419,371-456,414 |
|  |  |  |  |  | Chr | 5.8 | 589,224-595,051 |
|  | Chr | 34.4 | intact | 1,020,662-1,055,098 | Chr | 35.9 | 1,020,162-1,056,041 |
|  |  |  |  |  | Chr | 44.9 | 1,235,327-1,280,198 |
|  | Chr | 49.6 | intact | 1,230,648-1,280,198 | Chr | 5.3 | 1,256,908-1,262,240 |
|  |  |  |  |  | Chr | 66.0 | 1,749,992-1,815,936 |
|  | Chr | 67.6 | intact | 1,751,919-1,819,502 | Chr | 4.1 | 1,815,706-1,819,775 |
|  | Chr | 50.9 | intact | 2,249,571-2,300,473 | Chr | 37.0 | 2,249,287-2,286,319 |
| PF7 | Chr | 32.6 | intact | 74,228-106,868 | Chr | 61.5 | 47,001-108,517 |
|  | Chr | 38.7 | intact | 236,541-275,223 | Chr | 45.0 | 229,605-274,573 |
|  |  |  |  |  | Chr | 5.8 | 604,226-610,053 |
|  | Chr | 36.2 | intact | 1,035,690-1,071,914 | Chr | 36.5 | 1,035,190-1,071,657 |
|  | Chr | 67.5 | intact | 2,006,140-2,073,664 | Chr | 4.5 | 2,012,917-2,017,389 |
|  |  |  |  |  | Chr | 39.1 | 2,017,768-2,056,904 |
|  |  |  |  |  | Chr | 10.0 | 2,065,136-2,075,167 |
|  | Chr | 42.1 | intact | 2,252,565-2,294,708 | Chr | 53.3 | 2,240,395-2,293,719 |
| PF8 | Chr | 32.7 | intact | 73,962-106,665 | Chr | 61.6 | 46,735-108,314 |
|  | Chr | 38.7 | intact | 236,338-275,020 | Chr | 45.0 | 229,402-274,370 |
|  |  |  |  |  | Chr | 5.8 | 604,023-609,850 |
|  | Chr | 36.2 | intact | 1,035,487-1,071,711 | Chr | 36.5 | 1,034,987-1,071,454 |
|  | Chr | 67.5 | intact | 2,005,937-2,073,461 | Chr | 4.5 | 2,012,714-2,017,186 |
|  |  |  |  |  | Chr | 39.1 | 2,017,565-2,056,701 |
|  |  |  |  |  | Chr | 10.0 | 2,064,933-2,074,964 |
|  | Chr | 42.1 | intact | 2,252,362-2,294,505 | Chr | 53.3 | 2,240,192-2,293,516 |
| PF9 | Chr | 48.9 | intact | 45,656-94,604 | Chr | 50.2 | 46,852-97,078 |
|  | Chr | 47.9 | intact | 419,522-467,506 | Chr | 37.0 | 419,385-456,415 |
|  | Chr | 67.6 | intact | 886,187-953,774 | Chr | 65.7 | 887,673-953,355 |
|  |  |  |  |  | Chr | 16.3 | 1,118,583-1,134,887 |
|  | Chr | 43.4 | intact | 1,425,300-1,468,698 | Chr | 44.9 | 1,425,507-1,470,374 |
|  | Chr | 39.9 | intact | 1,645,062-1,685,036 | Chr | 35.9 | 1,647,726-1,683,627 |
|  |  |  |  |  | Chr | 5.8 | 2,110,693-2,116,520 |
|  | Chr | 37.7 | intact | 2,249,633-2,287,326 | Chr | 37.0 | 2,249,332-2,286,369 |
| PF10 | Chr | 48.9 | intact | 45,656-94,590 | Chr | 50.2 | 46,852-97,064 |
|  | Chr | 36.6 | intact | 419,510-456,116 | Chr | 37.0 | 419,373-456,420 |
|  |  |  |  |  | Chr | 5.8 | 589,233-595,060 |
|  | Chr | 34.4 | intact | 1,020,716-1,055,152 | Chr | 35.9 | 1,020,216-1,056,095 |
|  | Chr | 49.5 | intact | 1,230,697-1,280,245 | Chr | 44.1 | 1,236,162-1,280,245 |
|  |  |  |  |  | Chr | 14.1 | 1,571,498-1,585,612 |
|  | Chr | 67.6 | intact | 1,751,968-1,819,555 | Chr | 66.0 | 1,749,971-1,815,989 |
|  | Chr | 50.9 | intact | 2,249,627-2,300,525 | Chr | 37.0 | 2,249,343-2,286,371 |
| PF11 | Chr | 34.9 | intact | 130,337-165,217 | Chr | 62.7 | 128,688-191,350 |
|  | Chr | 44.8 | intact | 863,868-908,644 | Chr | 35.6 | 872,410-907,992 |
|  |  |  |  |  | Chr | 5.8 | 1,237,653-1,243,480 |
|  | Chr | 36.2 | intact | 1,669,121-1,705,345 | Chr | 36.5 | 1,668,621-1,705,088 |
|  | Chr | 55.5 | intact | 2,180,720-2,236,255 | Chr | 56.9 | 2,181,358-2,238,211 |
|  | Chr | 44.7 | intact | 2,416,775-2,461,512 | Chr | 5.4 | 2,409,635-2,415,060 |
|  |  |  |  |  | Chr | 31.3 | 2,423,295-2,454,601 |
|  |  |  |  |  | Chr | 4.8 | 2,456,684-2,461,512 |
| PF12 | Chr | 48.9 | intact | 45,656-94,604 | Chr | 50.2 | 46,852-97,078 |
|  | Chr | 36.6 | intact | 419,519-456,118 | Chr | 37.0 | 419,382-456,422 |
|  |  |  |  |  | Chr | 5.8 | 589,236-595,063 |
|  | Chr | 34.4 | intact | 1,020,719-1,055,155 | Chr | 35.9 | 1,020,219-1,056,098 |
|  | Chr | 49.5 | intact | 1,230,706-1,280,259 | Chr | 44.9 | 1,235,385-1,280,259 |
|  |  |  |  |  | Chr | 14,1 | 1,571,510-1,585,624 |
|  | Chr | 67.6 | intact | 1,751,980-1,819,552 | Chr | 66.0 | 1,749,983-1,815,986 |
|  |  |  |  |  | Chr | 4.1 | 1,815,757-1,819,825 |
|  | Chr | 50.9 | intact | 2,249,617-2,300,508 | Chr | 37.0 | 2,249,333-2,286,354 |
| PF13 | Chr | 36.9 | intact | 612,513-649,391 | Chr | 14.1 | 81,893-96,007 |
|  |  |  |  |  | Chr | 5.4 | 267,404-272,828 |
|  |  |  |  |  | Chr | 15.8 | 611,978-627,763 |
|  |  |  |  |  | Chr | 7.1 | 635,548-642,657 |
|  | Chr | 63.1 | intact | 786,189-849,272 | Chr | 45.1 | 795,638-840,766 |
|  |  |  |  |  | Chr | 5.8 | 1,174,604-1,180,431 |
|  | Chr | 35.3 | intact | 1,774,309-1,809,608 | Chr | 37.4 | 1,765,776-1,803,192 |
|  | Chr | 58.9 | intact | 1,934,922-1,993,833 | Chr | 50.2 | 1,943,577-1,993,800 |
|  | P | 37.8 | intact | 1,849-39,626 | P | 38.1 | 1,461-39,593 |
|  | P | 10.7 | incomplete | 249,754-260,453 |  |  |  |
| PF14 | Chr | 48.9 | intact | 45,656-94,596 | Chr | 50.2 | 46,852-97,070 |
|  | Chr | 36.6 | intact | 419,514-456,117 | Chr | 37.0 | 419,377-456,421 |
|  |  |  |  |  | Chr | 5.8 | 589,218-595,045 |
|  | Chr | 34.4 | intact | 1,020,701-1,055,137 | Chr | 35.9 | 1,020,201-1,056,080 |
|  | Chr | 49.5 | intact | 1,230,685-1,280,232 | Chr | 44.9 | 1,235,364-1,280,232 |
|  |  |  |  |  | Chr | 14.1 | 1,571,483-1,585,597 |
|  | Chr | 67.6 | intact | 1,751,953-1,819,544 | Chr | 66.0 | 1,749,956-1,815,976 |
|  | Chr | 50.9 | intact | 2,249,620-2,300,519 | Chr | 37.0 | 2,249,336-2,286,365 |
| PF15 | Chr | 51.4 | intact | 45,354-96,794 | Chr | 45.8 | 46,697-92,510 |
|  |  |  |  |  | Chr | 5.8 | 425,562-431,389 |
|  | Chr | 35.2 | intact | 1,025,236-1,060,406 | Chr | 37.4 | 1,016,736-1,054,152 |
|  | Chr | 58.9 | intact | 1,185,849-1,244,754 | Chr | 48.9 | 1,194,537-1,243,412 |
|  |  |  |  |  | Chr | 14.1 | 1,536,053-1,550,167 |
|  |  |  |  |  | Chr | 8.9 | 1,721,564-1,730,437 |
|  | Chr | 36.9 | intact | 2,066,695-2,103,573 | Chr | 22.7 | 2,066,243-2,088,955 |
|  | Chr | 47.4 | intact | 2,249,762-2,297,201 | Chr | 46.1 | 2,249,854-2,295,984 |
| PF16 | Chr | 48.9 | intact | 45,656-94,596 | Chr | 47.4 | 46,852-94,240 |
|  | Chr | 36.6 | intact | 419,513-456,097 | Chr | 37.0 | 419,376-456,382 |
|  | Chr | 67.6 | intact | 886,158-953,745 | Chr | 65.5 | 887,801-953,326 |
|  |  |  |  |  | Chr | 16.3 | 1,118,554-1,134,858 |
|  | Chr | 43.4 | intact | 1,425,271-1,468,663 | Chr | 44.9 | 1,425,478-1,470,339 |
|  | Chr | 40.0 | intact | 1,645,027-1,685,001 | Chr | 35.9 | 1,647,691-1,683,592 |
|  |  |  |  |  | Chr | 5.8 | 2,110,657-2,116,484 |
|  | Chr | 51.0 | intact | 2,236,331-2,287,302 | Chr | 31.4 | 2,250,560-2,281,994 |
| PF17 | Chr | 32.7 | intact | 74,225-106,946 | Chr | 64.4 | 47,001-111,425 |
|  | Chr | 38.7 | intact | 236,658-275,320 | Chr | 37.4 | 237,280-274,670 |
|  |  |  |  |  | Chr | 4.3 | 282,280-286,619 |
|  |  |  |  |  | Chr | 5.8 | 604,301-610,128 |
|  | Chr | 36.2 | intact | 1,035,765-1,071,989 | Chr | 36.5 | 1,035,265-1,071,732 |
|  | Chr | 67.5 | intact | 2,006,109-2,073,613 | Chr | 4.5 | 2,012,886-2,017,358 |
|  |  |  |  |  | Chr | 39.1 | 2,017,737-2,056,852 |
|  |  |  |  |  | Chr | 6.3 | 2,061,638-2,069,380 |
|  | Chr | 42.1 | intact | 2,252,512-2,294,646 | Chr | 51.8 | 2,241,902-2,293,657 |
| PF18 | Chr | 48.9 | intact | 45,656-94,586 | Chr | 47.4 | 46,852-94,230 |
|  | Chr | 36.6 | intact | 419,506-456,103 | Chr | 26.3 | 419,369-445,708 |
|  |  |  |  |  | Chr | 5.8 | 589,214-595,041 |
|  | Chr | 34.4 | intact | 1,020,697-1,055,133 | Chr | 35.9 | 1,020,197-1,056,076 |
|  | Chr | 49.6 | intact | 1,230,683-1,280,236 | Chr | 44.9 | 1,235,362-1,280,236 |
|  |  |  |  |  | Chr | 14.1 | 1,571,487-1,585,601 |
|  | Chr | 59.6 | intact | 1,759,928-1,819,481 | Chr | 66.4 | 1,749,960-1,816,357 |
|  | Chr | 50.9 | intact | 2,249,599-2,300,501 | Chr | 12.0 | 2,250,772-2,262,776 |
| PF19 | Chr | 32.7 | intact | 74,223-106,943 | Chr | 61.6 | 47,001-108,592 |
|  | Chr | 38.7 | intact | 236,655-275,321 | Chr | 45.0 | 229,719-274,672 |
|  |  |  |  |  | Chr | 5.8 | 604,318-610,145 |
|  | Chr | 36.2 | intact | 1,035,782-1,072,006 | Chr | 36.5 | 1,035,282-1,071,749 |
|  | Chr | 67.8 | intact | 2,006,122-2,073,950 | Chr | 4.5 | 2,012,899-2,017,371 |
|  |  |  |  |  | Chr | 39.4 | 2,017,750-2,057,190 |
|  |  |  |  |  | Chr | 13.5 | 2,061,976-2,075,453 |
|  | Chr | 42.1 | intact | 2,252,849-2,294,989 | Chr | 51.5 | 2,242,451-2,294,000 |

Chr: chromosome, P: plasmid.
